# Platelet Serotonin Level Predicts Survival in Amyotrophic Lateral Sclerosis

**DOI:** 10.1371/journal.pone.0013346

**Published:** 2010-10-13

**Authors:** Luc Dupuis, Odile Spreux-Varoquaux, Gilbert Bensimon, Philippe Jullien, Lucette Lacomblez, François Salachas, Gaëlle Bruneteau, Pierre-François Pradat, Jean-Philippe Loeffler, Vincent Meininger

**Affiliations:** 1 INSERM U692, Strasbourg, France; 2 Université de Strasbourg, Strasbourg, France; 3 Faculté de Médecine Paris-Ile de France-Ouest, Paris, France; 4 Université de Versailles Saint-Quentin-en-Yvelines, Versailles, France; 5 Centre Hospitalier Versailles, Le Chesnay, France; 6 Université Pierre et Marie Curie, Paris, France; 7 UMR 7211, CNRS, Paris, France; 8 Service de Pharmacologie Clinique, Hôpital de la Pitié-Salpêtrière (AP-HP), Paris, France; 9 Département des Maladies du Système Nerveux, Centre Référent Maladie Rare SLA Hôpital de la Pitié-Salpêtrière (AP-HP), Paris, France; 10 INSERM U678, Paris, France; Tufts University, United States of America

## Abstract

**Background:**

Amyotrophic lateral sclerosis (ALS) is a life-threatening neurodegenerative disease involving upper and lower motor neurons loss. Clinical features are highly variable among patients and there are currently few known disease-modifying factors underlying this heterogeneity. Serotonin is involved in a range of functions altered in ALS, including motor neuron excitability and energy metabolism. However, whether serotoninergic activity represents a disease modifier of ALS natural history remains unknown.

**Methodology:**

Platelet and plasma unconjugated concentrations of serotonin and plasma 5-HIAA, the major serotonin metabolite, levels were measured using HPLC with coulometric detection in a cohort of 85 patients with ALS all followed-up until death and compared to a control group of 29 subjects.

**Principal Findings:**

Platelet serotonin levels were significantly decreased in ALS patients. Platelet serotonin levels did not correlate with disease duration but were positively correlated with survival of the patients. Univariate Cox model analysis showed a 57% decreased risk of death for patients with platelet serotonin levels in the normal range relative to patients with abnormally low platelet serotonin (p = 0.0195). This protective effect remained significant after adjustment with age, gender or site of onset in multivariate analysis. Plasma unconjugated serotonin and 5-HIAA levels were unchanged in ALS patients compared to controls and did not correlate with clinical parameters.

**Conclusions/Significance:**

The positive correlation between platelet serotonin levels and survival strongly suggests that serotonin influences the course of ALS disease.

## Introduction

Amyotrophic lateral sclerosis (ALS) is a neurodegenerative disorder affecting both lower motor neurons (LMN) and upper motor neurons (UMN), and leads to death within 2 to 5 years of onset. Clinical features and progression are highly heterogeneous among patients (for review see [1]). There are currently few identified disease-modifying factors accounting for this heterogeneity. Among these, energy metabolism abnormalities have been shown in ALS patients [2], [3], [4], [5] and their potential contribution to the course of the disease has been stressed [6], but little is known about the factors triggering these abnormalities.

The neurotransmitter serotonin is involved in a range of functions altered in ALS, including motor neuron excitability and energy metabolism (reviewed in [7], [8], [9]). Early studies on very limited numbers of patients suggested that the levels of serotonin and its metabolite are decreased in brain tissues of ALS patient's post-mortem [10], [11], [12]. Moreover, recent imaging studies have shown decreased binding of serotonin 1A (5-HT1A) ligands in ALS raphe and cortex [13]. However, definite evidence that serotonin itself is modified in ALS is lacking and whether serotonergic activity is a disease modifier of ALS natural history remains unknown. Since serotonergic neurons and platelets express similar serotonin related enzymes and receptor, alterations in central serotonin are likely to be reflected in platelet serotonin levels [14], [15], [16]. In this study we aimed to study the relationships between circulating serotonin and survival in a cohort of ALS patients.

## Results

### Decreased platelet serotonin levels in ALS patients

The demographic data of ALS patients (Table 1) are in close accordance with previous reports thus showing that the patients included in the present study are representative of the ALS population. Age and sex ratio of ALS and control groups did not differ (Table 1). Levels of platelet serotonin were significantly lower in ALS patients (median [range] ng/mL: 78.4 [5.2–232.1]) as compared to controls (median [range] ng/mL: 110.6 [41.4–239.6], p = 0.0003) with a global 30% decrease in ALS patients (Table 2). Normal values of the laboratory range from 60 to 200 ng/mL (N = 69 patients) [17], [18]. Compared to normal value of the laboratory, 31% of ALS patients displayed values below the normal range, as compared to 6% of controls. Plasma unconjugated serotonin and 5-HIAA remained unchanged. The decreased platelet serotonin levels were found in patients with either bulbar or spinal onset (Table 3). Levels of plasma unconjugated serotonin were decreased in bulbar, but not in spinal onset patients compared to controls (Table 3). 5-HIAA plasma levels were similar in controls and in patients whatever was the site of onset, and the molar ratio between 5-HIAA and platelet serotonin (an index of MAO-A activity) was significantly increased in ALS patients compared to matched controls (Table 2), and this was mostly due to bulbar onset patients. This difference between bulbar and spinal onset patients was not due to a difference in nutritional status between these two groups since BMI did not differ significantly (bulbar onset patients: 24.6±3.5; spinal onset patients: 24.7±3.4; p>0.05).

**Table 1:** Demographic parameters in ALS and control groups.

Item (+/−SD)	ALS patients	Controls
N	85	29
Mean age at blood sampling (years)	61.65±10.5	61.0±14.4
Sex ratio (M/F)	1.24	1.18
Bulbar site of onset (bulbar/spinal)	34% (29/56)	
Age at onset (years)	60.3±9.9	
Age at death (years)	64.20±9.8	
Survival (months)	45.9±40.7	
BMI (kg/m^2^)	24.7±3.5	

**Table 2:** Serotonin levels in ALS and control groups.

Variables (mean ± SD; n)	ALS patients	Controls	P value
Platelet serotonin (ng/mL)	83.4±50.3; 82	124.3±47.7; 29	0.0003
Plasma unconjugated serotonin (ng/mL)	1.35±1.78; 82	1.41±1.20; 29	NS
Plasma 5-HIAA (ng/mL)	7.35±4.07; 72	7.71±2.38; 29	NS
5-HIAA/platelet serotonin molar ratio	0.14±0.20; 70	0.07±0.04; 29	0.012

Groups were compared using Mann-Whitney test and the p-value is shown.

**Table 3:** Serotonin levels in ALS patients as a function of site of onset.

Item (+/−SD; n)	ALS patients (Bulbar onset)	ALS patients (Spinal onset)	Controls
Platelet serotonin (ng/mL)	76.9±47.5; 28**	86.8±51.7; 54**	124.3±47.7; 29
Plasma unconjugated serotonin (ng/mL)	0.88±1.08; 26*	1.56±2.00; 56	1.41±1.20; 29
Plasma 5-HIAA (ng/mL)	8.00±3.31; 23	7.04±4.37; 49	7.71±2.38; 29
5-HIAA/platelet serotonin molar ratio	0.21±0.30; 22*	0.12±0.13; 49	0.07±0.04; 29

Groups were compared using Kruskal Wallis test. *, p<0.05; **, p<0.01.

### Decreased platelet serotonin is predictive of worsened survival of ALS patients

There was a significant decrease of platelet serotonin with increasing age at blood sampling in ALS patients (R squared  = 0.053, p = 0.0365) but not in controls (R squared  = 0.004825, p = 0.72). In the ALS patients group, there was no relationship of platelet serotonin with the length of disease prior to sampling (R squared  = 0.016324 p = 0.2527). Platelet serotonin levels showed no correlation with either site of onset, sex, Norris score slopes, initial BMI or rate of BMI loss. Neither plasma serotonin nor 5-HIAA levels correlated with any of the clinical parameters.

**Figure 1:**
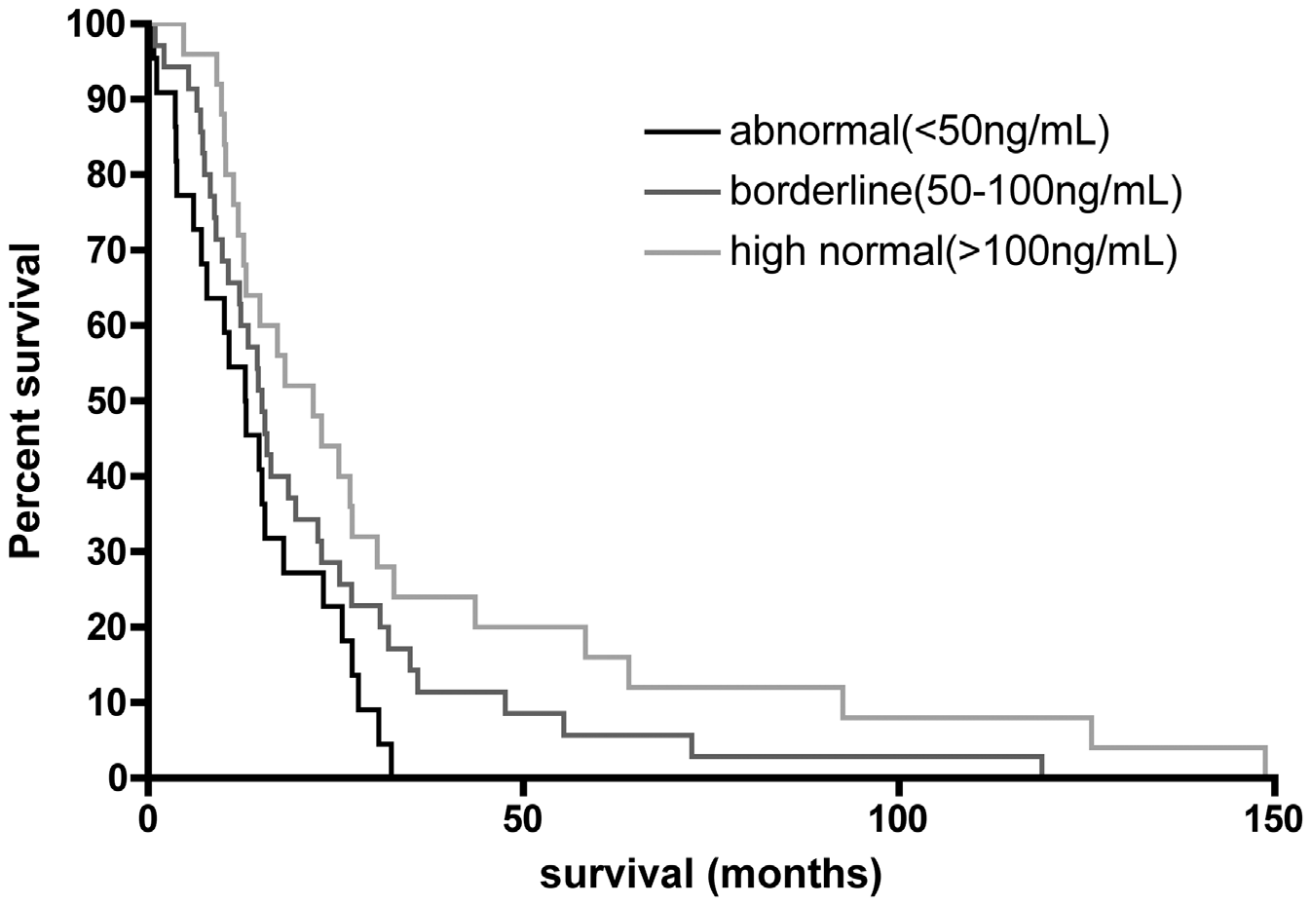
Correlation between platelet serotonin levels and survival in patients with amyotrophic lateral sclerosis (ALS). Kaplan-Meyer survival curves of patients with ALS stratified according to their platelet serotonin levels. Survival was calculated as the time period between blood sampling and death. The black line represents patients with ALS having abnormal serotonin values (serotonin <50 ng/mL, n = 22 patients); light grey line represents patients with high normal values (serotonin >100 ng/mL i.e. median of control group, n = 25 patients), and light black line patients with borderline values (50–100 ng/mL, n = 35 patients). Group comparison shows a statistically significant difference (p = 0.024 by the log-rank).

Overall median survival, calculated from time of sampling to death, was 34 months [range: 1-149]. Univariate Cox model analysis of survival showed a significant increase of risk of death with increasing age (Cox model, RR [95%CI] (per year)  = 1.028 [1.004–1.055], p = 0.0210) and significant decrease of the risk of death with increasing platelet serotonin levels (Cox model, RR [95%CI] (per ng/mL)  = 0.994 [0.989–0.999], p = 0.0195). We broke down patient population in 3 groups according to platelet serotonin levels. Values were considered as in a high normal range when superior to 100 ng/mL (i.e. approximately the mean of controls), in an abnormal low range when inferior to 50 ng/mL, and in a borderline range between 50 and 100 ng/mL. This analysis showed a good linearity of the platelet serotonin levels on survival (Figure 1). Patients in the high normal values range showed a 57% decreased risk of death relative to the abnormal low values range and 35% relative to the borderline one (p = 0.023 by the log-rank test, Figure). Platelet serotonin remained a significant independent predictor of survival following adjustment on known prognostic variables such as age, or site of onset or gender (RR [95%CI] (per ng/mL)  = 0.995 [0.990–0.999], p = 0.046; RR [95%CI] (per ng/mL)  = 0.984 [0.989–0.999], p = 0.020 and RR [95%CI] (per ng/mL)  = 0.995 [0.989–0.999], p = 0.026 respectively).

## Discussion

Our study provides two important results, (i) platelet serotonin levels are significantly decreased in ALS patients relative to matched controls, and (ii) platelet serotonin level is a significant predictor of survival independent of age.

We first show that platelet serotonin levels are globally decreased in ALS patients, with about one third of ALS patients displaying levels of platelet serotonin below the normal range.

Platelets are usually considered as an easily accessible model of central serotonergic neurons due to similarities in enzymatic and receptor equipments [14], [16]. Thus, the decreased platelet serotonin levels could mirror what has been reported in ALS patients, i.e., a spinal cord decrease in both serotonin and its metabolite as well as a decreased binding of 5-HT1A ligand in the brain [9], [10], [11], [12], [13]. Patients with bulbar onset displayed a more profound impairment of the serotonergic system since plasma unconjugated serotonin levels were also decreased in this population, in contrast with patients with spinal onset. This is in line with a more pronounced loss of cortical 5-HT1A binding observed in bulbar onset patients [13].

The causes of serotonin decrease remain elusive. Number of neurologic and psychiatric conditions, including Alzheimer's disease [19], are associated with decreases of the serotonergic system either centrally or in platelets or both. A similar decrease in serotonin has been described in frontotemporal dementia [20], which is now thought to form a continuum with ALS [21]. Platelet serotonin decrease was also found in depression, although to a lesser extent [18], [22], [23] and in violent suicide attempters [24]. Consistent with a pathogenic role of serotonin decrease in ALS, number of studies showed that depression in ALS worsens disease severity [25], [26], [27]. In all, our current study supports the existence of a marked impairment of the serotonergic system in a significant proportion of ALS patients. It should be pointed out that decreased levels of platelet serotonin have been reported in many different pathological or debilitating conditions, notably in psychiatric and neurologic diseases, and thus platelet serotonin levels in ALS, as observed decreased here, should not be considered as a specific biomarker of ALS. Another limitation of our study is that serotonin levels were assayed in blood, at one single time point. Future studies should be longitudinal and measure brain-derived serotonin in the CSF.

The second major result of this study is that platelet serotonin levels correlated positively with survival, as calculated from time of sampling to death. Numbers of studies have shown that serotonin levels drop with age [28]. Since age is a known modifier of ALS survival, [29] it was critical to determine whether the observed correlation was related to age and not to the disease process. In our study, platelet serotonin did not correlate with age in controls. This is likely to be due to the relatively narrow age range of patients, since the most important variations of serotonin with age occur in the first two decades of life [30]. In the ALS population, after adjustment with age in a multivariate Cox model, platelet serotonin remained a significant predictor of survival, thus showing that the correlation between platelet serotonin and survival unlikely results from an effect of age on platelet serotonin levels. Another potential confounding factor in studies with serotonin is drug interactions. The observed correlations were not due to such drug interactions since patients taking serotonin reuptake inhibitors, antidepressants or any other serotonin related medications were excluded from the study. It should be noted that in this study, serotonin was measured at one single time point, when disease had already been diagnosed. Thus, serotonin decrease might be either preexisting disease onset and be associated with an intrinsic variation in serotonin metabolism, or be the consequence of the disease process itself. The observed positive correlation might, in this latter case be due to an upstream mechanism able to modify both ALS course and serotonin levels. In this case, serotonin decrease would be a surrogate marker of a crucial, still unknown, pathogenic event. In the current absence of longitudinal studies or of genetic studies on serotonin-related genes, it remains impossible to discriminate between these two hypothesis.

Previous research in animal models of ALS however supports that serotonin might directly modulate onset and survival in ALS. The administration of serotonin precursor 5-hydroxytryptophan delayed onset and mortality in a transgenic mice ALS model [31]. The mechanisms underlying the potential protection by serotonin in ALS clearly deserve further investigation. It seems unlikely that the potential positive effect of serotonin on survival is related to the excitatory action on motor neurons [7], [8], [32] since increased serotonin potentially exacerbates a glutamate-evoked excitotoxicity [9]. A more attractive hypothesis is a relationship between serotonin and energy metabolism. Serotonin modulates energy homeostasis through complex and still incompletely characterized mechanisms [33]. A large number of ALS patients as well as transgenic ALS mice show increased energy expenditure [3], [4], [5], [34], a phenotype which appears analogous to the effects of a chronic depletion in brain-derived serotonin [35]. Future studies should focus on the potential relationships between serotonin and energy metabolism in ALS.

Whether serotonin directly modulates disease course or constitute a surrogate marker of another crucial pathogenic event, our results show that platelet serotonin correlates with survival in ALS. To our knowledge, few biological factors have been demonstrated to be associated with survival in ALS. Among them are the increase of LDL/HDL ratio [6], of plasma ApoE [36], the polymorphisms in the *kifap3* gene [37] or more recently CSF glial markers [38]. In the current state of knowledge, our results do not indicate that serotonin replacement therapy could be useful for patients, but rather that investigations focused on the serotonergic system in ALS are warranted.

## Materials and Methods

### Ethics statement

Patients were informed of the aim of this non-interventional research and gave their written informed consent. The institutional review board of Salpêtrière hospital approved the research. Control patients gave their written informed consent for participating in this study.

### Patients

Blood samples were collected from 90 patients followed at the Pitié Salpetrière hospital (Paris, France) between 1994 and 1995. All patients met the El Escorial World Federation of Neurology criteria for the diagnosis of definite or probable ALS at the time of blood sampling. Patients underwent blood sampling after diagnosis and before riluzole treatment. Control samples were collected from 11 healthy volunteers and 18 patients hospitalized for orthopaedic surgery. There was no difference in any of the measured parameters between healthy volunteers and orthopaedic patients and they were thus considered as a single control group. Controls were age and sex matched and medical examinations excluded neurodegenerative, inflammatory, psychiatric (in particular depression and food intake disorders, such as anorexia) or neoplasic disorders of the nervous system. All blood samples were taken as a part of routine laboratory evaluations and were collected after a 12 h fasting period, with care taken to avoid possible medications and possible diets and dietary problems known to interfere with serotonin levels. For controls undergoing orthopaedic surgery, blood samples were taken before any surgery to avoid for the potential biases due to surgery or immobilization. All individuals included in the study, either patients or controls, did not used serotonin reuptake inhibitors or any medication with known interaction with serotonin (*e.g.* antidepressants). Death was documented by death certificate or written letter from relatives or physicians. Documented time of death was obtained for all patients but 5 who were lost to follow-up and had no information after sampling visit. Database was closed at 1st October 2009 and these 5 patients were excluded from the study. Survival was defined as time from blood sampling to death. Disease duration was calculated as the time interval between first symptoms and blood sampling. These values were calculated in months. Body mass index (BMI) was calculated at each visit and BMI loss was calculated using a linear regression model. Bulbar and Limbs Norris scale scores were assessed at sampling visit and at each subsequent visit.

### Biochemical procedures

Circulating serotonin was measured in serum; while plasma unconjugated serotonin and 5-HIAA levels were measured in plasma. Platelet serotonin, which corresponds to about 98% of total circulating serotonin, was calculated as the difference between circulating and plasma unconjugated serotonin concentrations. The molar ratio between platelet serotonin and 5-HIAA levels was calculated and used as an indirect index of MAO-A activity. All the patients and the control subjects fasted overnight. Samples of blood were drawn from the anterocubital vein between 7AM and 10AM. Five millilitres of blood were collected into Vacutainer tubes with no additive for circulating 5-HT, with citrate for plasma unconjugated serotonin and with dry heparin for plasma 5-HIAA levels. Serum and plasma were separated by centrifugation at 3000 g and stored at –80°C until further analysis without additional thawing. The results were unrelated to storing time. The three parameters were determined within 6 months after blood sampling by high-performance liquid chromatography with coulometric detection according to three different analytical procedures previously described [39], [40]. The minimum quantifiable level of serotonin was 0.05 ng/mL in plasma and serum. The day-to-day variations were 2.0%, 3.0% and 2.7% for 25, 100 and 300 ng/mL respectively (n = 8) for serum serotonin and 8.6%, 7.0% and 5.6% for 0.5, 5 and 10 ng/mL plasma serotonin levels (n = 8). For plasma 5-HIAA assays, the minimum quantifiable level was 1 ng/mL with the day-to-day coefficient of variation of 14% and 11.2% for 6 and 21 ng/mL (n = 10) respectively.

### Statistical analysis

Data are expressed as mean ± SD and/or median [range].

Based on normal values of the laboratory (mean [95% CI]: 92.7 [83.4–101.9] ng/ml) [17], [18] we calculated that we could detect at least a 26% change in patients (n = 85) as compared to controls (n = 29) with 81% power and p-alpha (2-tailed) = 0.05, by the T test. For group comparisons (ALS *vs.* controls), we performed non-parametric Mann-Whitney tests. For comparisons of three groups (control *vs*. ALS patients with spinal onset or bulbar onset), we used Kruskal Wallis tests. Correlations were performed using the nonparametric Spearman's rank correlation test.

Univariate and multivariate Cox model analysis were performed to assess the effects of candidate variables on survival. To assess the linearity of platelet serotonin levels on survival, patient population was broken-down in 3 groups according to (i) high normal values (cut-off > median of control group = 100 ng/mL i.e. circa 550 nM, 25 patients), (ii) abnormal low values (cut-off below the normal range <50 ng/mL i.e. circa 280 nM, 22 patients) and (iii) borderline values (50–100 ng/mL, 35 patients). Survival curves for the three groups were compared by the Mantel Cox (log rank) statistic. Data were analysed using JMP software (version 8.0; SAS Institute Inc., Cary, NC), and statistical threshold for statistical significance set at p (α 2-tailed) <0.05.
